# Measuring Population Sodium Intake: A Review of Methods

**DOI:** 10.3390/nu6114651

**Published:** 2014-10-28

**Authors:** Rachael M. McLean

**Affiliations:** Departments of Preventive and Social Medicine/Departments of Human Nutrition, University of Otago, PO Box 56, Dunedin 9054, New Zealand; E-Mail: rachael.mclean@otago.ac.nz; Tel.: +64-3-479-9428; Fax: +64-3-474-7641

**Keywords:** dietary sodium, population, urine, epidemiology, monitoring

## Abstract

Reduction of population sodium intake has been identified as a key initiative for reduction of Non-Communicable Disease. Monitoring of population sodium intake must accompany public health initiatives aimed at sodium reduction. A number of different methods for estimating dietary sodium intake are currently in use. Dietary assessment is time consuming and often under-estimates intake due to under-reporting and difficulties quantifying sodium concentration in recipes, and discretionary salt. Twenty-four hour urinary collection (widely considered to be the most accurate method) is also burdensome and is limited by under-collection and lack of suitable methodology to accurately identify incomplete samples. Spot urine sampling has recently been identified as a convenient and affordable alternative, but remains highly controversial as a means of monitoring population intake. Studies suggest that while spot urinary sodium is a poor predictor of 24-h excretion in individuals, it may provide population estimates adequate for monitoring. Further research is needed into the accuracy and suitability of spot urine collection in different populations as a means of monitoring sodium intake.

## 1. Introduction

The World Health Organization (WHO) Global Action Plan for the Prevention and Control of Non-Communicable Diseases (NCDs) 2013–2020 identifies nine key targets for the reduction of chronic disease, including “a 30% relative reduction in mean population intake of salt/sodium” [1]. This target differs from, but is consistent with previous WHO statements on dietary sodium intake. In 2006, WHO identified a population mean intake of sodium of <2000 mg (5 g salt) as optimal [2], and in 2012, following two systematic reviews [3,4], an optimal individual level of dietary sodium intake was similarly identified as <2000 mg/day for adults with proportional lower levels for children [5]. This shift to a focus on a relative reduction rather than absolute level reflects the observation that worldwide, most populations have a mean intake that considerably exceeds that which is considered optimal. In 2010 the global mean sodium intake (based on analysis of intakes across 66 countries) was estimated to be 3.95 g/day, and was higher than WHO recommended levels in almost all countries [6]. The focus on relative reduction is also consistent with the observed linear relationship between sodium intake and blood pressure, with no apparent threshold below (or above) which the effect is attenuated [5]. A gradual reduction in population sodium intake is likely to be more achievable for countries, and so a focus on percentage reduction is a more pragmatic approach.

## 2. Monitoring

Essential to reporting on this target is regular population monitoring of dietary sodium intake. Many (particularly high income) countries already have high quality established systems of nutrient intake monitoring, and will find reporting on this target is relatively easily accommodated into existing monitoring systems. Other countries with limited resources or without existing nutrition monitoring will find this more challenging.

A number of different ways of measuring dietary sodium intake are currently available, including dietary and urinary assessment. There are challenges associated with the measurement of dietary sodium that make precise quantification of intake difficult. Both inter-individual and intra-individual variability of sodium intake is high, due to high variability of usual dietary patterns [7]. Measuring population sodium intake however does not require a valid estimate of each individual’s mean intake, but rather a valid estimate of the range and frequency of intakes across the population. Therefore it is important that the sample is representative of the population (thereby minimising bias) and that the methods used provide a valid estimate of mean population level intake. An adequate sample size will account for inter-individual variability.

### 2.1. Dietary Assessment

Dietary recall and weighed diet records are widely used methods of assessing nutrient intakes, and are labour intensive for both participants and researchers. Subjects may change their behaviour when collecting dietary information prospectively (such as in a weighed diet record), and under-reporting of intake, both generally and of particular foods and nutrients has been reported in the literature [8,9]. Sodium intake is highly correlated with total energy intake, due to its inclusion in a wide variety of foods and meals [10]. Under-reporting of energy (and therefore sodium) intake is common, and has been shown to be greater for those with higher body mass index (BMI) scores [11]. With an increasing proportion of the population in many high- and middle-income countries becoming overweight or obese, under-reporting is likely to be highly prevalent. Particular issues arise with quantification of dietary sodium in dietary assessment: the sodium content in recipes for both processed and home-cooked foods is highly variable, and discretionary salt use (in home cooking or at the table) is difficult to quantify and often not included in standard dietary surveys. Different patterns of sodium consumption in different populations must be accounted for. For example, in countries with a western style diet, most intake (75%–80%) is from sodium contained in processed food, with only around 10% estimated to be added in the home in cooking or at the table. In other countries (such as China), the majority is added in home cooking through salt or sauces [12,13]. Dietary surveys are often considered unsuitable for estimating population sodium intake as they tend to under-estimate intake due to omission or difficulty quantifying discretionary salt added in the home. A comparison of 24 h recall using the United States Department of Agriculture (USDA) Automated Multiple-Pass Method used in the National Health and Nutrition Examination Survey (NHANES) with 24 h urine in healthy volunteers aged 30–69 years found the mean (95% CI) (calculated as the ratio of reported dietary sodium intake from 24-h recall to 24-h urinary sodium/0.86 assuming that 86% of sodium ingested is excreted in the urine) of 24 h recall was 0.93 (0.89, 0.97) for men and 0.90 (0.87, 0.94) for women, suggesting that the USDA Automated Multiple-Pass Method is a valid method for assessing dietary sodium intake [14]. Sodium in medicines may also be an important source of sodium intake which may be missed in dietary surveys [15], although one study indicated that intake from dietary supplements was very low [14].

Food frequency questionnaires (FFQs) have also been used to estimate sodium intake [16]. FFQs are useful as they assess intake over a longer period than dietary surveys, and potentially overcome problems associated with the high day-to-day variability of intake, however precise quantification of daily intake is extremely difficult. It is unlikely that FFQs would be able to quantify intake accurately enough for population monitoring. One calibration study using the mean six 24 h urine collections over a 12 month period as the reference gold standard measure, showed that a seven-day diet record was a more reliable estimate of intake than the FFQ when it came to quantifying intake [17].

While the validity of dietary assessment tools is variable, they are essential for informing public health interventions for dietary sodium reduction, as they enable identification of sources of sodium intake. Identification of foods associated with high intake in different populations and cultural groups is essential to inform public health interventions based on reformulation of processed foods, and consumer education, and changing dietary practices. Dietary assessment also allows the linking of sodium intake with dietary patterns or intake of other nutrients (such as potassium) associated with disease related outcomes in order to inform public health interventions (see for example [13,18]). Furthermore, assessment of sodium intake within a wider dietary survey allows for calculation of energy-adjusted intake, favoured by many epidemiologists. Adjustment for total energy intake is used in epidemiological studies to control for confounding by other factors that may be associated with total energy intake (such as sex, body size, and physical activity levels). Sodium intake is highly correlated with energy intake so that if the dietary assessment tool is significantly underestimating total food intake (and therefore total energy intake) then there will be comparable underestimating of dietary sodium from food sources [19].

### 2.2. 24 h Urine Collection

24 h urine collection is widely regarded as the gold standard method for assessment of intake, and is often used as the measure by which to compare and validate other methods of sodium intake assessment. As approximately 90% of ingested sodium is excreted in the urine over the same period an accurate 24 h urine collection reflects intake reliably. Variable losses also occur through sweat and feces, and have been estimated to be around 10% under normal conditions, but may be greater in hot climates, or among populations who are highly physically active. Seasonal variability has also been reported with the proportion of dietary sodium excreted in the urine noted to be lower in summer compared to winter [20]. Twenty four hour urine collection has been used to assess population sodium intake in the landmark INTERSALT study which estimated dietary sodium intake in 52 population groups in 32 countries, and, more recently in the United Kingdom to evaluate the success of its population sodium reduction strategy [21].

Although 24 h urinary assessment of sodium is likely to be more valid than dietary assessment, collection of 24 h urine involves considerable burden for participants, which may influence response rates and collection in representative population surveys. Although some surveys report reasonable response rates (for example 43% in a United Kingdom survey in 2005 [22] and 43%–57% in a Slovene population in 2007 [23]) other response rates are reported to be as low as 10% [24]. Low response rates may lead to bias in population surveys, as respondents are likely to be more health-conscious than non-respondents, although the extent to which this applies in surveys relating to assessment of dietary sodium intake has not been established. A recent Australian population survey showed that estimates of sodium consumption did not differ substantively between participants randomly selected and those who volunteered, suggesting that estimates of population sodium consumption obtained from volunteers was valid in this context [25].

Due to the difficulties associated with the accurate collection of a complete 24 h collection, both under-collection and over-collection of samples has been reported [26]. Various methods exist to identify collections that may be incomplete, however there is no method which can discriminate with certainty between complete and incomplete samples [27]. *Para*-aminobenzoic acid (PABA) has been widely used to assess completeness of urine collections, including in recent UK population surveys. The use of PABA requires subjects to take a tablet three times spaced evenly throughout the day on the day of collection with morning, daytime and evening meals. PABA is excreted almost completely in the urine over the same time period. Urine collections with less than a pre-specified cut off (commonly 85%) are classified as incomplete [28]. Incomplete collections are traditionally disregarded, although with as many as 71% of collections in one study deemed to be incomplete, methods for adjusting results to compensate for assumed under-collection have been suggested [28]. A number of other limitations of use of PABA to determine completeness of urine collections have been reported including declining rates of excretion with increasing age, non-adherence to the relatively inflexible PABA dosage regime, and potential interaction with other medications [29].

Other methods used to assess the completeness of 24 h urine collections include assessment of 24 h creatinine excretion, 24 h urine volume, self-report or a combination of these factors. Twenty-four hour creatinine excretion is associated with body weight, age, sex and protein intake. A number of models have been determined to predict 24 h excretion based on calculations based on age, sex and weight, however these have unacceptably low sensitivity for detecting all incomplete collections [30]. A very low urine volume is indicative of under-collection, however this is unlikely to accurately identify all incomplete samples in a population survey. Participants may also be asked to report any missed or spilled collections [31]. Some researchers have used a combination of methods. For example in the INTERSALT study both the start and end of collection period took place in the clinic with times recorded and participants were asked to report if they had missed any collections [32]. 24 h collections were rejected if participants reported losing ‘more than a few drops’ of urine or if urine volume was low (<250 mL) [33]. A study of healthy adult volunteers aged 18–39 years assessed completeness of 24 h urine by volume (≥500 mL), self reported collection period (>20 h) and self reported missing or spilled urine, and 24 h creatinine excretion [34]. Using this method only 85% returned a complete 24 h urine collection [34]. Under-collection can be high in population surveys. For example only (52.3%) of the 24-h samples collected in a UK population sample were estimated to be complete with the PABA recovery method, although adjustment for incomplete samples enabled a greater proportion to be used in the survey [22]. In a more recent survey conduced in England in 2011, 23% of 24 h urine collections were classified as incomplete using the PABA recovery method and were excluded from further analysis [35].

It is clear therefore that although 24 h urine collection is used in validation studies as the gold standard method, it is labour intensive for both participants and researchers and samples are often collected inaccurately. The potential bias introduced by low response rates and undetected under-collection must be acknowledged when used in population samples. Estimates based on urinary assessment do not provide information on dietary sources of sodium intake necessary to inform public health interventions. However, a clear advantage of 24 h urine collection is its portability across different populations, dietary patterns and food cultures, which allows for valid international comparisons.

### 2.3. Spot Urine

Much recent attention has been focussed on the possibility of using a single spot urine to estimate 24 h urinary sodium excretion. Spot urines have been widely used to assess nutritional and clinical biomarkers in other settings [36,37]. The use of a single spot urine collection has many potential advantages: it can be relatively easily incorporated into a wider population health and or nutrition surveys, and is able to be collected in a single encounter, thereby bypassing need for multiple visits. Spot urine samples are easily collected and stored without potential for under or over collection. Typically, spot or random urine samples are collected as a single pass while participants visit a survey centre, and stored in a small airtight container. Samples may be frozen, depending on length of time to analysis. Other parameters used in conversion formulae must be measured and/or recorded such as age, sex, weight, height, and urinary creatinine and potassium (depending on which formula is used) in order to interpret urinary sodium results [31]. Spot urine sampling is therefore potentially a practical and affordable alternative to 24 h excretion in population surveys [24]. However their validity for estimating population sodium intake is still under investigation, and remains highly controversial [38].

Several different formulae have been proposed to convert spot urine sodium into an estimate of 24 h excretion, using spot urine sodium: creatinine ratio as a means to control for urinary concentration. Tanaka *et al*. [39] proposed a formula based on analysis of spot and 24 h urine samples from 591 Japanese participants (295 men and 296 women) from the INTERSALT study; and Kawasaki *et al*. [40] conducted a similar analysis using a second morning voiding urine specimen from 159 Japanese participants (78 men and 81 women). More recently a formula has been published which is derived calibration of spot and 24 h urine samples from the Western INTERSALT study including 2841 male and 2852 female North American and European adults 20–59 years of age [41,42]. A simpler formula has been proposed by the Pan American Health Organization [31], which is based on one developed for use in the National Health and Nutrition Survey (NHANES) to predict chemical exposure [43]. With much current interest in this topic it seems likely that others will also emerge.

A number of validation studies have been published using comparison of spot with measured 24 h excretion as the gold standard measure. Two main questions arise:

1. Is an estimate based on a single spot urine a valid reflection of an individual’s mean 24 h excretion?
2. Is an estimate based on the mean of a single spot urine collection in a population, a valid reflection of the mean sodium intake of that population?

Spot urinary sodium concentration (even when sodium: creatinine ratio is used to account for urinary concentration) is likely to represent sodium intake over a short time period (only a few hours). Diurnal variation of sodium has been described, with over night samples reported to have lower sodium concentrations than those collected during the day [34]. Spot urine samples therefore are likely to show even greater intra-individual variability of sodium concentration than 24 h collections, especially if the samples are collected at different (and varying) times of the day, as is likely in large population surveys. Wang *et al*. reported large within person variances for timed spot urine samples (ranging from 21% to 41% of mean excretions) in the spot specimens. These were larger than for the 24 h urines, which had 16%–29% of the mean excretion within person variation. Repeated spot urine sodium: creatinine ratio had poor reliability in a New Zealand population sample with an intra-class correlation of only 0.25 [44]. A single spot urine will not therefore estimate an individual’s mean sodium intake.

Many studies have compared spot with 24 h on same day to assess whether a single spot urine can accurately estimate 24 h excretion on that day. A review of validation studies conducted in 2012 showed correlation coefficients of ranging from 0.17 to 0.94 when comparing an estimate based on spot urine collection with a 24 h urine collection over the same period [45]. This review concluded that a single spot urine is not a good reflection of 24 h excretion over the same time period [45]. Similarly, assessments using the mean difference ratio of Bland and Altman (usually considered to be a better method of assessing agreement between two methods of measurement than correlation) also show spot urine to be a poor predictor of 24 h excretion in individuals. For example a comparison in a New Zealand population showed that indicated that for 95% of cases, the ratio of the estimated 24 h urinary sodium using the INTERSALT formula will be between 0.46 and 2.56 times the measured 24 h urinary sodium for the same 24 h time period [44].

However many studies have shown that the mean population intake derived from spot urine sampling using predictive equations to estimate individual mean 24 h excretion approximates the mean 24 h excretion of that population, indicating that it may be a useful tool for monitoring population sodium intake. A study in a New Zealand sample of healthy volunteers who provided spot urine and a 24-h urine samples showed that estimates using the INTERSALT formula provided reasonably accurate estimates of key population indicators (mean, range and proportion above nutrient reference values) that were close to those of the measured 24-h urine excretion [44]. A recent review of 19 studies, including 6803 participants which compared population means from collected spot and 24 h urine sodium excretion concluded that spot urine sodium is likely to be suitable for estimating population level estimates [46]. This study suggested that estimates using the INTERSALT formula may perform better than Tanaka or Kawasaki estimates across a range of values of 24 h excretion in a range of different population groups [46]. A validation study in multi-ethnic populations in Britain and Italy using estimates calculated using the Tanaka formula showed limited agreement between estimates based on spot and 24 h urinary excretion. In particular, the validity of estimates using spot urine differed between men and women, and in different ethnic groups, suggesting that spot urine estimates have limited usefulness in comparing sodium intake in different ethnic groups [47].

The use of spot urine estimates of 24 h excretion is being considered as a means of monitoring dietary sodium intake in low income countries where 24 h urine sampling may be logistically difficult [48]. It also shows promise in the interpretation of population-based settings. For example Pfeiffer *et al*. have estimated 24 h excretion in US adults aged 20–59 years participating in NHANES surveys between 1988 and 2010 using estimates derived from a convenience sample of casual/random spot urine samples using the INTERSALT formula [41]. They demonstrated a small but statistically significant increase in estimated mean sodium intake (both with and without adjustment for age, sex, ethnicity) over the period, suggesting that monitoring population sodium intake using spot urine sampling in population surveys may be a valid [41]. In general, estimates based on spot urine were comparable with estimates based on NHANES dietary intake and 24 h urine from other studies over the period [41]. This finding is contrasts with a study by Bernstein and Willet which examined trends in sodium 24 h excretion in US between 1957 and 2003 showing no significant change in sodium excretion over this period [49]. This apparent discrepancy between the two studies may arise may because the 24 h urine samples are taken from different surveys using different populations and different methods of recruitment and sampling However further research is needed using spot urine to estimate population mean intake in different populations before this method becomes widely accepted. A further limitation of studies to date has been the limited age range included, with few of the validation studies using participants over the age of 65 years of age; the age group most at risk of cardiovascular disease outcomes. Baseline 24 h urine assessment has been recommended [31], and validation studies may be recommended to assess which (if any) of the published formulae is valid for use in different population groups prior to monitoring population sodium intake using spot urine sampling.

Several countries have recently measured spot urine sodium as part of population surveys, including Australia, New Zealand. However the lack of a well validated and internationally accepted formula for conversion to estimates of 24 h excretion has meant that these results have not yet been reported [24,50]. While several formulae have been proposed, a single formula has not been accepted for widespread international use. It is plausible that different formulae may be suitable for estimation in different ethnic or population groups, and validation studies in different populations will be required. It is likely however that provided a single formula is used over time, any bias inherent to the use of that formula would remain relatively constant and a percentage change in population intake would be demonstrated, consistent with the Global Target.

## 3. Epidemiological Studies

It must be emphasized that while the use of spot urine sampling to estimate population sodium intake shows some promise, spot urine sampling has been shown to be inaccurate as a measure of individual sodium intake in clinical settings or epidemiological studies [44,51]. Due to substantial variability in sodium intake over time up to 10, 24 h assessments (based on 24 h urine or dietary assessment) are required to accurately estimate an individual’s mean sodium intake [7,52]. For example the INTERMAP study used two timed 24 h urine collections (using several techniques to assess completeness of collection) as well as two 24-h recalls to estimate sodium intake of participants [27]. A well designed and validated food frequency questionnaire may also be useful for identifying whether individuals are high- or low-sodium consumers in clinical or epidemiological settings [53], however assessments based on a single spot urine sample are likely to be completely invalid in this context.

## 4. Conclusions

Monitoring of population sodium intake is essential for compliance with the WHO target of a 30% relative reduction in mean population sodium intake. A number of different methods estimating dietary sodium are currently in use. Dietary assessment (diet records or diet recall) is labour intensive and often under-estimates intake due to under-reporting and difficulties quantifying sodium concentration in a variety of recipes, as well as discretionary salt intake. Dietary assessment does however enable identification of important dietary sources of sodium, which can inform public health interventions to lower sodium intake. 24 h urinary collection (widely considered to be the most accurate method) is also burdensome and is limited by under-collection and lack of suitable methodology to accurately identify incomplete samples. Spot urine sampling is potentially a convenient and afffordable alternative. Studies suggest that while spot urinary sodium is a poor predictor of 24 h excretion in indviduals, it may in the future provide population estimates adequate for monitoring as part of broader population surveys. However, there are still a number of questions about reliability of spot urine collections as a means of monitoring population changes. Therefore it is recommended that in those providing spot urine that a sub-sample also perform 24 h urine collections to enable the development of valid estimating equations that can provide a robust mean population estimate of sodium intake from spot collections. Regardless, it is important that the assessment methods are consistent to demonstrate trends over time. Whilst there are always going to be challenges in relation to finding a perfect dietary assessment method [19], further research is needed into how best to monitor population salt intake, especially in low income countries.
